# The effect of clinical and psychosocial factors on quality of life in COPD: a cross-sectional assessment using a mediation analysis approach

**DOI:** 10.3389/fpsyt.2026.1756291

**Published:** 2026-02-06

**Authors:** Selda Günaydın, Meltem Hazel Şimşek, Şaban Melih Şimşek

**Affiliations:** 1 Department of Chest Diseases, Faculty of Medicine, Giresun University, Giresun, Türkiye; 2 Department of Psychiatry, Faculty of Medicine, Giresun University, Giresun, Türkiye

**Keywords:** chronic obstructive pulmonary disease (COPD), emotion regulation, mediation analysis, perceived social support, psychosocial variables, quality of life

## Abstract

**Objective:**

This study aimed to examine the relative contributions of clinical severity indicators and psychosocial factors—including depression, anxiety, stress, emotion regulation difficulties, and perceived social support—on quality of life in patients with Chronic Obstructive Pulmonary Disease (COPD), and to test their mediating mechanisms within an integrated model.

**Methods:**

A total of 120 patients with COPD were assessed using spirometry, dyspnea, and symptom severity measures (mMRC, CAT), psychological assessments (DASS-21, DERS-16, MSPSS), and the WHOQOL-BREF. Mediation models using the PROCESS macro (Model 4) for SPSS were applied to evaluate the pathways linking clinical severity and psychosocial factors to quality-of-life domains.

**Results:**

Among 120 participants diagnosed with COPD, disease stages were found to be GOLD A: 25.8%, GOLD B: 35.8%, and GOLD E: 38.3%. Clinical severity measures (CAT, mMRC) were moderately correlated with both psychological symptoms and emotion-regulation difficulties (p < 0.05). CAT had a significant indirect adverse effect on physical quality of life via emotion regulation difficulties [b = –0.174, 95% CI (–0.303, –0.069)]. Perceived social support positively predicted physical quality of life via lower depressive symptoms (b = 0.52, 95% CI (0.06, 1.10)). Stress was indirectly associated with poorer psychological quality of life through reduced social support [b = –0.191, 95% CI (–0.363, –0.042)].

**Conclusion:**

Psychosocial mechanisms substantially contribute to quality-of-life impairment in COPD and operate alongside traditional clinical indicators. Emotion-regulation difficulties and social support emerge as key psychological pathways linking symptom severity and quality-of-life outcomes, suggesting that comprehensive COPD management should integrate psychosocial intervention strategies.

## 1 Introduction

Chronic Obstructive Pulmonary Disease (COPD) is a chronic lung disease characterized by irreversible abnormalities in the airways and/or alveoli of the lungs, often leading to progressive airflow limitation. The disease manifests with chronic respiratory symptoms, including shortness of breath, cough, and sputum production. COPD typically develops after many years of smoking, exposure to air pollution, dust, gases, and chemicals (1). The diagnosis of COPD is generally made based on clinical findings and spirometry. It is supported by imaging and additional laboratory findings (2). Staging in the Global Initiative for Chronic Obstructive Lung Disease (GOLD) is based on forced expiratory volume in one second (FEV_1_), dyspnea score, symptom burden, and exacerbation frequency in the last 12 months (3).

Socioeconomic factors such as poverty, malnutrition, and access to healthcare are among the risks for the severity of COPD, as are psychosocial factors including stress, mental health problems, and social isolation (4, 5). COPD is a significant public health problem worldwide and is among the leading causes of mortality. It is currently the fourth leading cause of mortality worldwide and is projected to become the third leading cause of mortality in middle-income countries by 2030 (6). COPD not only restricts people’s respiratory functions but also negatively affects their daily functioning, social roles, emotional functions, and general well-being (7).

Many studies have shown that quality of life decreases with increasing clinical stage of COPD (8, 9). In fact, quality of life has become one of the main criteria affecting the success of treatment in these patients (10). Therefore, in patients diagnosed with COPD, the sole aim of treatment is not to prolong life, but also to control symptoms, prevent exacerbations, and improve quality of life by preserving functional capacity (11). However, as the stage of COPD increases, the frequency of exacerbations, hospitalizations, and comorbidities also increases. All of these factors also increase the psychosocial and emotional burden. All of these factors significantly reduce the quality of life in patients with COPD (12). Studies have also reported that objective lung function measures, such as FEV1, partially explain patients’ quality-of-life levels (13). This suggests that focusing only on clinical and physiological indicators in the management of COPD and quality of life will be insufficient.

The basic physiological symptoms of COPD not only lead to physical limitations but also to severe and uncontrolled emotional stress (14). Mental disorders, particularly anxiety and depression, are much more common in patients with COPD than in the general population. The literature reports that the prevalence of anxiety in patients with COPD is 10–37%, while the prevalence of depression is 13–57% (15, 16). These prevalences increase as disease severity increases. Depression rates have been reported to exceed 50% in advanced-stage patients who are dependent on oxygen therapy and experience frequent exacerbations. (17). These mental problems seen in patients with COPD have been associated with poor adherence to treatment, limitations in daily activities, increased healthcare visits, and thus a significant decrease in quality of life (18). In fact, some studies have reported that the quality of life in patients with COPD is related to the severity of depression rather than the physical severity of the disease (19).

Another psychosocial factor that may have an impact on the management of COPD and the quality of life of these patients is perceived social support. This factor reflects the individual’s belief that they can receive adequate support from their social environment when needed (20). Higher perceived social support in patients with COPD reduces the risk of depression and anxiety and improves quality of life. Furthermore, increased treatment adherence and self-care behaviors have been associated with reduced symptom severity (21, 22). This has been linked to individuals with a high perception of social support being better able to cope with the stress and emotional burden of COPD, reducing the negative impact of depression and anxiety on quality of life (23).

Another psychological impact of a COPD diagnosis is difficulty regulating emotions. Difficulties in Emotion Regulation (DER) refers to the difficulty an individual has in accurately recognizing, accepting, and appropriately managing the emotions they experience. This concept is associated with a decline in effective coping skills in the face of emotional intensity or stressful life events (24). DER has been reported to be frequently observed in individuals with chronic illnesses. This has been attributed to the emotional stress, burden of illness, and decreased quality of life brought on by the disease (25). However, detailed studies are needed regarding the relationship between DER and COPD.

All of these suggests that the factors affecting quality of life in patients with COPD are multidimensional and cannot be explained only by respiratory dysfunction. Studies that examine respiratory clinical parameters together with psychosocial variables such as depression, anxiety, stress, and perceived social support in patients with COPD and comprehensively examine their relative effects on quality of life are limited. The current study aims to make a unique contribution to the literature as one of the holistic approaches that examines respiratory clinical parameters and psychosocial factors that determine quality of life in COPD within the same model, assesses the mediating role of psychological variables, and aims to reveal the protective effect of social support.

The hypotheses of the current study are:

H1: Greater symptom burden (CAT scores) and dyspnea severity (mMRC scores) are associated with lower quality of life in the physical and psychological domains of the WHOQOL-BREF.

H2: Greater symptom burden (CAT scores) and dyspnea severity (mMRC scores) are associated with higher levels of psychosocial variables, including depression, anxiety, and stress.

H3: Higher perceived social support is associated with lower psychosocial variables and higher quality of life.

H4a: Difficulties in emotion regulation (DERS-16) mediate the relationship between symptom burden (CAT scores) and physical quality of life.

H4b: Depression mediates the relationship between perceived social support and physical quality of life.

H4c: Perceived social support mediates the relationship between stress levels and psychological quality of life.

## 2 Materials and methods

### 2.1 Study design and sample

The study has a cross-sectional design. Sample size estimation was performed using G*Power software. Assuming a type I error rate of 5%, a study power of 95%, and a medium effect size (0.25), the minimum required sample size was calculated as 73 participants (26).

However, given that the study included multiple mediation analyses, which generally require larger sample sizes to detect indirect effects reliably, and that it followed a cross-sectional design with recruitment conducted within a predefined time frame, the target sample size was increased. In addition, a larger sample enabled subgroup analyses by GOLD COPD stage (A, B, and E). Patients were recruited consecutively from the outpatient clinic between April 2025 and October 2025. For mediation analyses, bias-corrected bootstrap procedures with 5,000 resamples were applied, and indirect effects were interpreted based on 95% confidence intervals (27).

During the recruitment period, 152 patients diagnosed with COPD presented to the Giresun Training and Research Hospital Chest Diseases Outpatient Clinic. Of these, 16 patients were excluded due to additional physical illnesses and comorbid chest pathologies, 12 due to inability to complete spirometry testing, and 4 due to psychiatric comorbidities that could interfere with questionnaire validity (e.g., psychotic disorders, bipolar disorder, neurocognitive disorders, or substance use disorders, excluding nicotine dependence). The remaining 120 individuals constituted the final study sample. Patients diagnosed with COPD by a pulmonologist according to the Global Initiative for Chronic Obstructive Lung Disease (GOLD) 2024 guidelines were included as participants (3). Disease severity was staged by the pulmonologist according to the expected FEV1 percentage in accordance with the GOLD 2024 guideline, and the Modified Medical Research Council (mMRC) dyspnea scale and the COPD Assessment Test (CAT) were used to determine clinical severity.

Exclusion criteria for our study included the presence of other obstructive or restrictive lung diseases such as asthma, bronchiectasis, interstitial lung disease, or active lung infection. Patients who were also in an acute COPD exacerbation, those who had received high-dose systemic corticosteroids within the past month, and those with severe systemic diseases such as active malignancy or advanced cardiac, hepatic, or renal failure were also excluded from the study. Those with cognitive impairments that prevented completion of the questionnaires, those who were pregnant or breastfeeding, those with psychiatric comorbidities, and those who were unable to complete spirometry were also excluded.

### 2.2 Data collection tools

#### 2.2.1 Sociodemographic and clinical data form

This form includes basic sociodemographic information such as age, gender, and occupation. Clinical data such as COPD duration, pack-years of smoking, comorbidities, medications used, and the number of attacks in the past year are also recorded.

#### 2.2.2 Clinical parameters/objective measurements

Spirometry was used to assess the patients’ respiratory status, and FEV_1_ (%) values were recorded. Disease severity was classified according to the GOLD criteria. Dyspnea level was measured using the mMRC dyspnea scale. This scale ranges from 0 to 4 points, with a score of ≥2 considered significant dyspnea. Disease symptom burden was also measured using the CAT. A total score of ≥10 indicates a high symptom level.

#### 2.2.3 Depression, Anxiety, and Stress Scale—21

This scale was used to measure depression, anxiety, and stress levels in the sample. This scale was developed by Henry and Crawford (2005) (28). The scale has a 4-point Likert-type response format. It consists of three subscales: depression, anxiety, and stress. These subscales consist of seven items, and the scores are summed and multiplied by two. Higher scores reflect a more negative mood. The Turkish version was adapted by Yılmaz, Boz, and Arslan (2017) (29). Internal consistency coefficients for the Turkish version were reported as 0.90 for the depression subscale, 0.82 for anxiety, and 0.88 for stress.

#### 2.2.4 Multidimensional Scale of Perceived Social Support

This scale was developed by Zimet and colleagues (1988) (30). The study measured patients’ perceived social support from various sources. The MSPSS consists of 12 items and is a 7-point Likert-type scale. It consists of three subscales: Family Support, Friend Support, and Perceived Support from a Significant Other. Each subscale contains four items. High scores on the scale indicate high perceived social support. The Turkish version was adapted by Eker et al. (2001) (31). The internal consistency coefficient (Cronbach’s alpha) of the Turkish form was 0.89 for the total scale and 0.85–0.91 for the sub-dimensions.

#### 2.2.5 Difficulties in Emotion Regulation Scale—16

This scale was developed by Bjureberg and colleagues (2016) as a short form of the original 36-item DERS (32). It is a 16-item, 5-point Likert-type self-report scale. Higher scores indicate increased difficulty regulating emotions. The Turkish adaptation was conducted by Yiğit et al. (2019) (33). The internal consistency coefficient of the Turkish version of this form was reported as 0.92 for the total scale and 0.78–0.91 for the subscales.

#### 2.2.6 World Health Organization Quality of Life—Short Form

This scale was used to assess the sample’s general quality of life. The scale was developed by the World Health Organization Quality of Life Group (WHOQOL Group, 1998) (34). It assesses an individual’s quality of life across four basic dimensions: physical, psychological, social relations, and environmental. It contains 26 items in total. It is scored on a 5-point Likert-type scale. Scores for each domain are calculated by averaging across the domains, ranging from 4 to 20, with higher scores indicating a better quality of life. The Turkish validity and reliability study was conducted by Eser et al. (1999) (35). The factor structure of the Turkish form is consistent with the original form; internal consistency coefficients (Cronbach’s alpha) were found to range between 0.53 and 0.83 (physical: 0.83; psychological: 0.66; social relations: 0.53; environmental: 0.73).

Variable selection and their roles in the mediation models were based on the biopsychosocial framework of chronic obstructive pulmonary disease (COPD). Clinical symptom burden and dyspnea severity are recognized as key factors impacting health-related quality of life in COPD, while objective lung function measures explain quality of life only partially (8, 9). Psychological comorbidities, including depression, anxiety, stress, and difficulties in emotion regulation, have been shown to contribute to functional impairment and reduced quality of life, beyond physiological indicators alone (18, 19, 24). In addition, perceived social support has been identified as a protective psychosocial factor that influences quality of life by mitigating psychological distress and enhancing coping capacity (20, 21). Based on this theoretical background, separate mediation models were developed to explore indirect pathways by which emotion regulation difficulties, depressive symptoms, and perceived social support might clarify links between clinical severity, psychosocial distress, and quality-of-life domains.

### 2.3 Ethical approval

This study was approved by the Giresun Training and Research Hospital Ethics Committee (Decision No: (15) Date: (30/04/2025). Written informed consent was obtained from all participants. The study was conducted in accordance with the principles of the 2013 Declaration of Helsinki.

### 2.4 Statistical analysis

Statistical analyses were performed using SPSS Statistics version 21. Descriptive statistics were presented as frequencies and percentages for categorical variables, and as mean ± standard deviation or median with interquartile range for continuous variables, as appropriate. The normality of continuous variables was evaluated using skewness and kurtosis values as well as graphical methods (histograms and Q–Q plots). Group comparisons were conducted using Student’s *t*-test or the Mann–Whitney *U* test, depending on data distribution. Categorical variables were analyzed using the chi-square test. Correlation analyses were performed using Pearson or Spearman correlation coefficients, depending on the distributional properties. A two-tailed *p* value of < 0.05 was considered statistically significant.

Mediation analyses were conducted using a regression-based bootstrapping approach implemented with the PROCESS macro version 4.2 for SPSS (Model 4), developed by Hayes (36). Bias-corrected bootstrap confidence intervals were generated using 5,000 resamples, as this approach provides reliable estimates of indirect effects without requiring normality assumptions for the indirect effect (36–38). An indirect effect was considered statistically significant when the 95% confidence interval did not include zero.

Although all WHOQOL-BREF domains were assessed, only the physical and psychological domains were included in the mediation analyses. This decision was based on preliminary correlation analyses showing that these domains were significantly associated with the clinical and psychosocial variables specified in the study hypotheses. Mediation models involving the social and environmental domains did not yield significant indirect effects and were therefore not pursued further to avoid model overfitting.

Perceived social support (MSPSS) was examined in different mediation models depending on the theoretical role specified *a priori*. In models focusing on the protective role of social support, MSPSS was treated as an independent variable predicting quality-of-life outcomes via psychological distress. In contrast, in models examining the impact of psychological distress on quality of life, MSPSS was specified as a mediator reflecting perceived availability and adequacy of social support. These models were tested separately and were not combined within a single mediation framework. All mediation models were adjusted for age, sex, smoking status, comorbidity status (presence of ≥1 comorbid condition), and GOLD stage.

The reporting of this study was prepared per the recommendations of the ‘Strengthening the Reporting of Observational Studies in Epidemiology (STROBE)’ guideline (39).

## 3 Results

Among the 120 patients diagnosed with COPD included in this study, the majority were male (91.7%). The majority of participants were married (91.7%), lived in urban areas (64.1%), and had low levels of education (81.7%). Most participants had comorbidities (77.8%), including hypertension (50%). When participants were classified according to GOLD COPD severity, the relationship was as follows: GOLD A 25.8%, GOLD B 35.8%, and GOLD E 38.3% (**Table 1**).

**Table 1:** Characteristics of participants (n: 120).

Characteristics	n (%)	Characteristics		n (%)
Gender		Educational Status		
Female	10 (8.3)	Uneducated		4 (3.3)
Male	110 (91.7)	Elementary School		98 (81.7)
Residency		High School		13 (10.8)
Rural	43 (35.9)	University		5 (4.2)
Urban	77 (64.1)	Comorbidities (n:120) *		
Marital Status		Any Comorbidity (Yes)		85 (70.8)
Married	110 (91.7)	Any Comorbidity (No)		35 (29.2)
Single/Divorced/	10 (8.3)	Hypertension		60 (50)
Smoking Status		Diabetes Mellitus Type 2		17 (14.2)
Current	54 (45)	Coronary Artery Diseases		38 (31.7)
Ex	66 (55)	COPD Exacerbations in the past 12		
Grade of COPD		months		
GOLD A	31 (25.8)	0		70 (58.3)
GOLD B	43 (35.8)	1–2		22 (18.3)
GOLD E	46 (38.3)	3 and more		28 (23.3)
mMRC		Exacerbations requiring hospitalization in		
Grade 0	25 (20.8)	the last 12 months		
Grade 1	36 (30)	Yes		17 (14.2)
Grade 2	28 (23.3)	No		103 (85.8)
Grade 3	26 (21.7)			
Grade 4	5 (4.2)			
		Mean ± SD	%95 GI	
Age		66.48 ± 7.39	65.17 – 67.80	
Body Mass Index (BMI)		26.34 ± 4.65	25.53 – 27.13	
COPD duration		6.55 ± 5.71	5.55 – 7.61	
Smoking Duration (packet-year/P-Y)		40.99 ± 26.51	36.16 – 45.98	
FEV1 (%predicted)		59.65 ± 23.97	55.19 – 63.74	
FVC (%predicted)		58.23 ± 19.11	54.63 – 61.56	
COPD Assessment Test (CAT)		14.63 7.85	13.26 – 16.08	

Values are presented as mean ± standard deviation (SD) or number (%) unless otherwise indicated.

BMI: body mass index; COPD: Chronic Obstructive Pulmonary Disease; GOLD: Global Initiative for Chronic Obstructive Lung Disease; FEV1: forced expiratory volume in one second; FVC: forced vital capacity.

*Percentages for specific comorbid conditions (hypertension, diabetes mellitus, coronary artery disease) are calculated based on the total study population. Participants may have more than one comorbidity.

When the distributions of clinical and psychosocial indicators according to GOLD staging were examined in **Table 2**, significant differences were found in mMRC (p <0.001) and CAT scores (p <0.001), as well as in the anxiety (p = 0.008), depression (p <0.001), and stress (p = 0.002) subscales of the DASS-21, according to COPD stages.

**Table 2:** Group comparisons of clinical and psychosocial variables across COPD stages (GOLD classification).

Variables	GOLD A (n:31)	GOLD B (n:43)	GOLD E (n:46)	*p*-value	*Post-Hoc*
mMRC	0.90 ± 0.87	1.12 ± 0.17	2.08 ± 1.15	<0.001	A<B=E*
CAT	6.38 ± 16.88	16.88 ± 5.50	18.08 ± 8.12	<0.001	A<B=E*
DERS-16	12.70 ± 9.58	16.11 ± 12.78	16.91 ± 12.15	0.288	
MSPSS					
Family	5.51 ± 1.33	5.20 ± 1.56	5.36 ± 1.50	0.679	
Friends	5.12 ± 1.72	5.00 ± 1.63	4.92 ± 1.63	0.877	
Significant Other	5.08 ± 1.78	5.05 ± 1.65	4.94 ± 1.64	0.927	
Total	5.23 ± 1.47	5.09 ± 1.51	5.07 ± 1.40	0.878	
DASS-21					
Anxiety	9.87 ± 8.03	16.0 ± 8.64	14.82 ± 8.77	0.008	A<B=E**
Depression	9.54 ± 7.46	17.02 ± 8.93	17.91 ± 8.72	<0.001	A<B=E**
Stress	9.41 ± 7.93	17.53 ± 10.97	15.73 ± 9.21	0.002	A<B=E**
WHOQOL-BREF					
Physical Health	50.80 ± 12.04	51.88 ± 10.69	48.50 ± 12.23	0.381	
Psychological	54.43 ± 13.12	55.37 ± 12.24	53.39 ± 11.15	0.742	
Social Relationships	55.64 ± 15.67	63.22 ± 23.15	55.84 ± 16.05	0.119	
Environment	56.85 ± 14.10	58.86 ± 13.30	55.84 ± 13.39	0.569	
Overall quality of life	62.90 ± 14.15	60.17 ± 13.91	57.88 ± 16.21	0.351	
General health	61.08 ± 14.49	58.86 ± 13.92	55.02 ± 17.85	0.229	

ANOVA; *Tamhane’s T2; **Tukey.

CAT, COPD Assessment Test; DASS-21, Depression, Anxiety and Stress Scale-21; DERS-16, Difficulties in Emotion Regulation Scale-Brief Form; mMRC, Modified Medical Research Council Dyspnea Scale; MSPSS, Multidimensional Scale of Perceived Social Support; WHOQOL-BREF, World Health Organization Quality of Life Assessment.

This difference was determined to be due to the GOLD B and GOLD E groups compared to the GOLD A group, but no significant difference was found between the GOLD B and GOLD E groups. Details are presented in **Table 2**.

When the relationships between clinical symptom markers and psychosocial variables in COPD are examined in **Table 3**, FEV_1_, a spirometric measurement, was negatively correlated only with the depression subscale of the DASS-21 (r = –0.221, p = 0.015). CAT and mMRC scores showed moderate positive correlations with all DERS-16 and DASS-21 subscales (p < 0.05). In addition, CAT and mMRC scores showed a low-level negative correlation with the physical and general health subscales of the WHOQOL-BREF. Additionally, a low-level negative correlation was observed between CAT and the WHOQOL-BREF global quality-of-life assessment (r = –0.228, p = 0.012).

**Table 3:** Relationships between clinical markers of COPD and psychosocial factors.

Psychosocial variables	mMRC	CAT	FEV1 (%predicted)
DERS-16	R: 0.229 *p*: 0.012	R: 0.344 *p*:<0.001	R: −0.130 *p*:0.156
MSPSS			
Family	R: 0.008 *p*: 0.931	R: 0.065 *p*:0.479	R: −0.24 *p*: 0.794
Friends	R: 0.020 *p*: 0.832	R: 0.021 *p*:0.818	R: −0.001 *p*:0.993
Significant Other	R:0.045 *p*:0.622	R: 0.044 *p*:0.635	R: 0.034 *p*:0.713
Total	R:0.028 *p*:0.765	R: 0.047 *p*:0.61	R:0.005 *p*:0.960
DASS-21			
Anxiety	R: 0.213 *p*:0.02	R: 0.457 *p*:<0.001	R: −0.150 *p*:0.102
Depression	R: 0.269 *p*:0.003	R: 0.493 *p*:<0.001	R: −0.221 *p*:0.015
Stress	R: 0.215 *p*:0.017	R: 0.396 *p*:<0.001	R: −0.117 *p*:0.204
WHOQOL			
Physical Health	R: −0.207 *p*:0.023	R: −0.222 *p*:0.015	R: 0.083 *p*:0.368
Psychological	R: 0.034 *p*:0.714	R: −0.089 *p*:0.332	R: 0.056 *p*:0.541
Social Relationships	R: −0.101 *p*:0.273	R: −0.024 *p*:0.794	R: 0.038 *p*:0.678
Environment	R: −0.065 *p*:0.479	R: −0.081 *p*:0.377	R: 0.020 *p*: 0.824
Overall quality of life	R: −0.127 *p*:0.166	R: −0.228 *p*:0.012	R: 0.130 *p*:0.158
General health	R: −0.253 *p*:0.005	R: −0.245 *p*:0.007	R: 0.113 *p*:0.218

*Pearson’s

CAT, COPD Assessment Test; DERS-16, Difficulties in Emotion Regulation Scale-Brief Form; FEV1, forced expiratory volume in one second; mMRC, Modified Medical Research Council Dyspnea Scale; MSPSS, Multidimensional Scale of Perceived Social Support; WHOQOL, World Health Organization Quality of Life Assessment.

**Figure 1:**
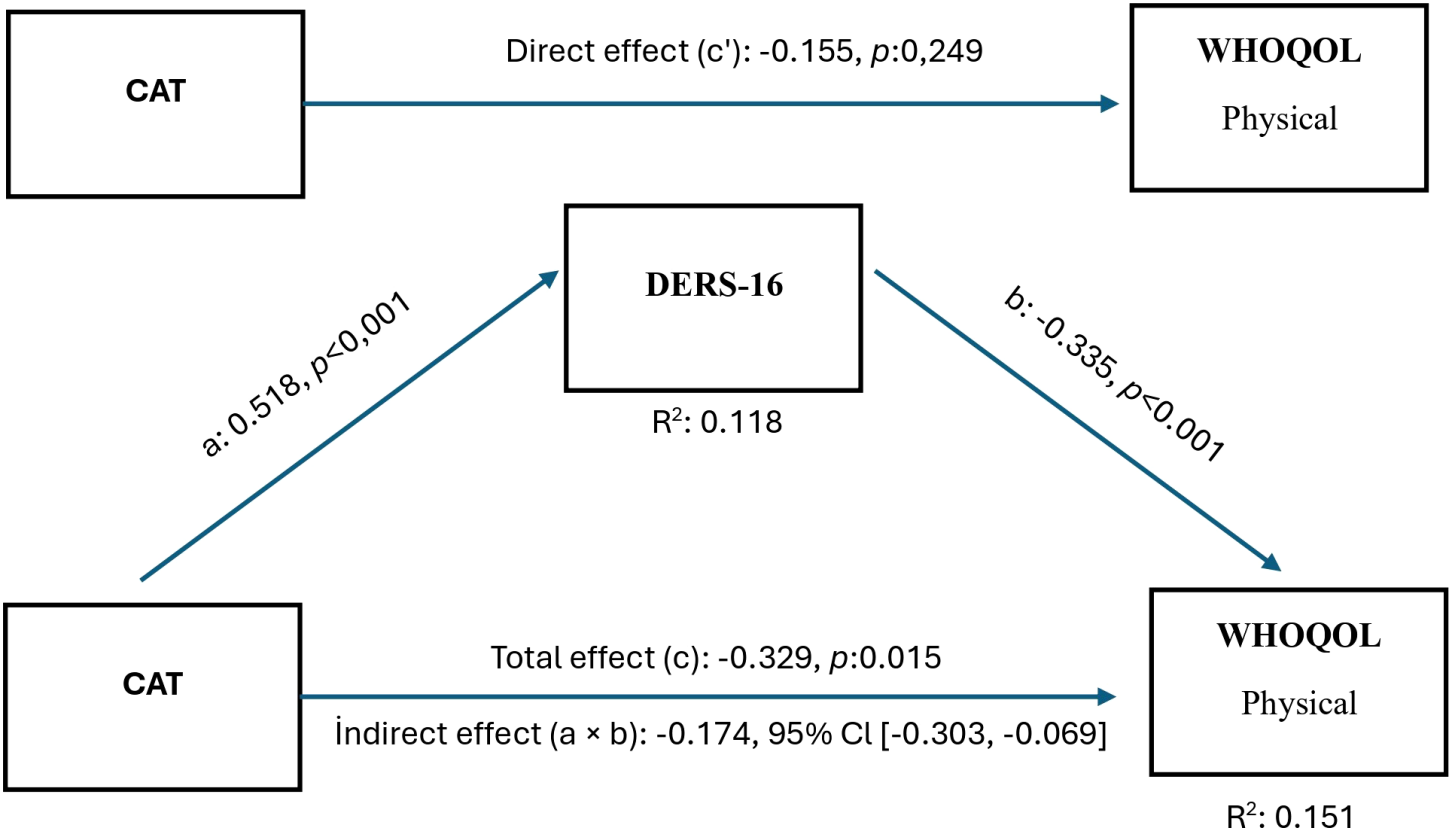
Mediation model for the association between COPD symptom burden (CAT) and WHOQOL-Physical domain via emotion regulation difficulties (DERS-16). Indirect pathway (a × b) indicates the mediating effect of emotion regulation difficulties. COPD stage was included as a covariate.

**Figure 1** shows the relationship between the CAT score and the WHOQOL-Physical subtype. While the direct effect of CAT on physical quality of life (c′ path) was not found to be significant, the indirect impact (a × b) via DERS-16 was found to be negative and important (a × b: −0.174, 95% CI (−0.303, −0.069)). Accordingly, as disease symptom severity increases, difficulty in emotion regulation increases, which reduces physical quality of life. This mediating effect was independent of COPD stage.

**Figure 2:**
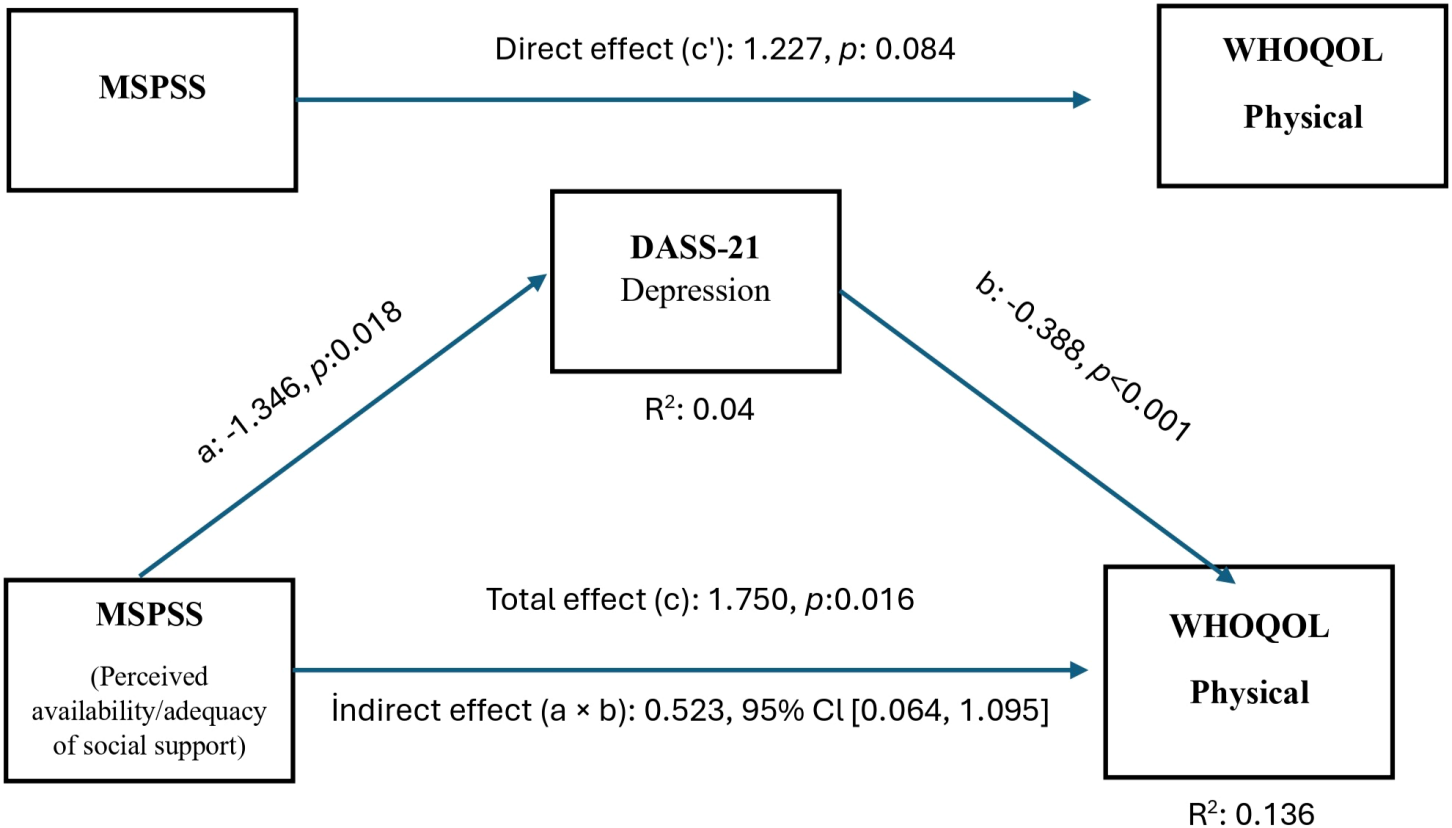
Mediation model showing the indirect association between perceived social support (MSPSS) and WHOQOL-Physical domain through depressive symptoms (DASS-21 Depression). Indirect pathway (a × b) reflects the mediating role of depression. COPD GOLD stage was controlled as a covariate.

**Figure 2** shows that the MSPSS score is positively correlated with the WHOQOL-Physical subdomain. However, according to the mediation analysis in Model 4, this relationship was determined to be indirect, not direct, through the DASS-21 Depression. Perceived social support was found to reduce depression levels (a = –1.35, p = 0.018), and a decrease in depression levels improved physical quality of life (b = –0.39, p <0.001). The indirect effect (a × b = 0.52, 95% CI (0.06, 1.10)) was found to be significant and positive. These results indicate that perceived social support improves quality of life in patients with COPD by reducing depressive symptoms. Furthermore, this mediation effect remained statistically significant after adjusting for age, sex, comorbidity status, smoking status, and GOLD stage.

**Figure 3:**
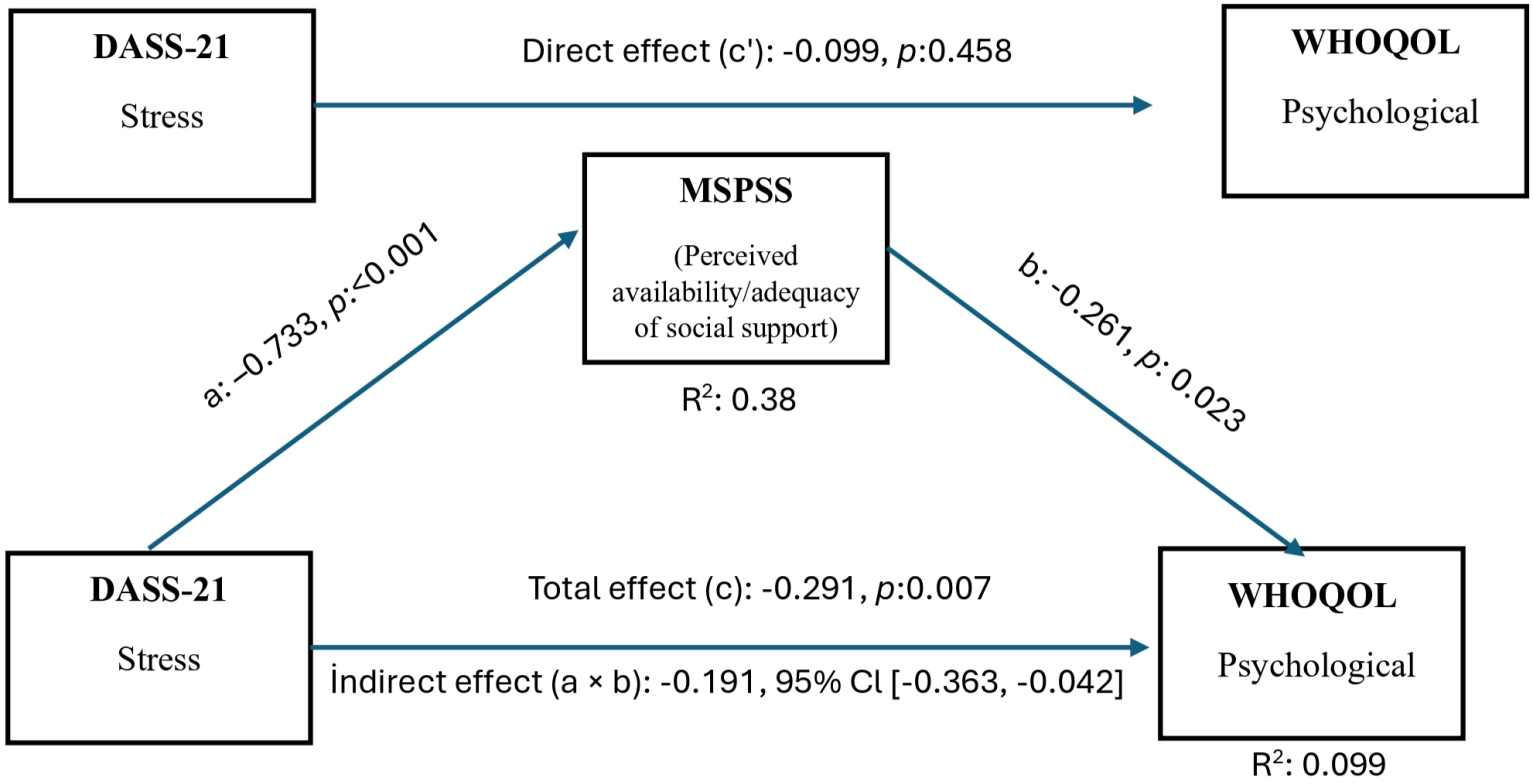
Mediation model illustrating the indirect association between stress levels (DASS-21 Stress) and WHOQOL-Psychological domain through perceived social support (MSPSS). Indirect pathway (a × b) represents the mediating effect of social support. COPD GOLD stage included as a covariate.

**Figure 3** shows that the DASS-21 stress score is negatively correlated with the WHOQOL-Psychological subdomain. According to Model 4 mediation analysis, this relationship was not significant via the direct (c′) path (p = 0.458). Still, the mediation effect of the MSPSS (a × b) was statistically significant and negative (95% CI (–0.363, –0.042)). According to this finding, higher stress levels are associated with lower perceived availability and adequacy of social support, which in turn is associated with poorer psychological quality of life. The total effect (c) was negative (p = 0.007). Furthermore, this mediation effect remained statistically significant after adjusting for age, sex, comorbidity status, smoking status, and GOLD stage).

## 4 Discussion

This study aimed to evaluate, in detail, the relationships between clinical symptom indicators of COPD and psychosocial factors, and their impact on quality of life. Our study showed that FEV1, an objective measure, did not directly and significantly affect quality of life, whereas symptom burden and dyspnea levels did. However, this was influenced considerably not only by clinical presentation but also by psychosocial symptoms. In particular, DER significantly mediated the relationship between disease severity and the physical quality-of-life subscale. Furthermore, perceived social support was seen as a protective factor that improved physical quality of life by reducing depression levels. Furthermore, emotional stress was found to both directly affect the psychological quality-of-life subscale and, through perceived social support, reduce psychological quality of life.

Many studies have observed that as COPD progresses, respiratory distress, physical limitations, and dependence on daily functions increase. Consequently, the likelihood of experiencing psychological symptoms also increases. (7). Similarly, in our study, depression, anxiety, and stress levels were found to be higher in the GOLD B and E groups compared to the GOLD A group. However, the number of studies investigating the relationships among clinical stage, DER, and perceived social support is quite limited. Our study findings suggest that DER and perceived social support may also constitute a psychosocial basis independent of clinical stage.

In this study, symptom burden and dyspnea scores were negatively correlated with the physical and general health subscales of quality of life. This suggests that symptom severity in COPD significantly negatively impacts individuals’ daily life performance and subjective assessment of their overall health. Literature has shown that as symptom severity increases in COPD, decreased walking performance, increased fatigue rate, avoidance of physical activities, and increased dependence on daily tasks develop. This directly impacts both individuals’ physical capacity and their perception of general health. (8, 12, 40).

Our study found that COPD symptom severity is positively correlated with DER and psychological symptoms. Studies have shown that depression, anxiety, and stress increase as COPD symptom severity increases (41). Among the reasons for this is that COPD-specific symptoms increase feelings of helplessness, fear, and anxiety in patients. Furthermore, systemic inflammation and chronic immune dysregulation in COPD play a role in the development of anxiety and depression. Factors such as loss of independence and social isolation brought on by the disease also increase the psychological burden. (42, 43). On the other hand, severe psychosocial variables have been associated with more respiratory symptoms, increased frequency of exacerbations, hospitalization, and risk of mortality (14). These results indicate that COPD requires a detailed evaluation of psychosocial factors, in addition to respiratory factors. Furthermore, to our knowledge, there are no studies investigating the relationship between COPD symptom severity and DER. However, in some chronic diseases other than COPD, DER has been reported to increase with increasing symptom severity or disease stage (44, 45).

A key finding of our study is that multidimensional symptom burden, as indicated by the CAT score, in patients with COPD indirectly reduces physical quality of life by increasing DER. This result suggests that increased symptom burden in COPD leads individuals to use maladaptive emotion regulation strategies more frequently. This indicates that the physical quality of life is not solely affected by respiratory parameters, but that DER may also be an important determinant. Similar to this finding in the literature, it has been reported that maladaptive emotion regulation strategies are used more frequently as disease severity increases in patients with coronary artery disease (45). On the other hand, although not specifically in COPD, it has been shown that DER causes a significant decrease in the psychological and social quality of life sub-dimensions in individuals with chronic diseases (46, 47). However, to our knowledge, no study has evaluated the mediating role of emotion regulation difficulties in the relationship between symptom burden and quality of life in COPD.

In our study, perceived social support was identified as one of the psychosocial factors affecting the physical quality of life of Patients with COPD. However, this effect was not direct, but was achieved indirectly by reducing depression levels. This suggests that perceived social support in patients with COPD improves quality of life by having a protective effect on mood. Studies have shown that depression is one of the most critical determinants of quality of life in Patients with COPD. Depressive symptoms can impair physical quality of life independently of respiratory symptoms (48). Perceived social support has been strongly shown to reduce emotional burden and depressive symptoms in people with COPD (49). A large-sample multicenter study showed that social support indirectly affects quality of life through depression. (50). All this information indicates that perceived social support can be considered a basic protective mechanism against depression in COPD.

Another notable finding of our study is that stress levels indirectly impair psychological quality of life in individuals with COPD through perceived social support. The findings of this model indicate that as stress levels increase, perceptions of social support decrease, and this decrease in perceived social support is associated with a significant decline in psychological quality of life. High stress levels in patients with COPD may negatively influence individuals’ perceptions of the availability and adequacy of social support, even in the absence of measurable changes in actual social interactions (49). Low perception of social support in these patients was found to be associated with higher anxiety, loneliness, hopelessness, and decreased psychological and physical quality of life. (22). Consistent with these findings, our results underscore the importance of strengthening social support systems to maintain psychological well-being in this population.

In our study, psychosocial burden was associated with clinical severity; however, this relationship did not follow a strictly linear pattern across GOLD stages. Notably, a marked increase in psychosocial variables was observed between GOLD A and GOLD B, suggesting a threshold effect rather than a continuous progression. In contrast, CAT scores demonstrated a more sensitive and consistent association with psychosocial distress, indicating that symptom-based measures may better reflect the subjective psychological impact of COPD than GOLD staging alone. These findings highlight the importance of incorporating patient-reported outcomes into evaluations of psychosocial functioning in COPD.

Most participants in this study were men, which should be kept in mind when looking at the results. Previous studies show that men and women can differ in how they handle emotions, manage stress, and seek support from others (51, 52). Men with COPD might not always report emotional problems or may use other ways to deal with them, unlike women (53). This could affect how symptom severity, emotion regulation, social support, and quality of life relate to each other in our findings. The larger number of men in the study matches the trend seen in Turkey, especially in the Black Sea region, where men are more likely to have and be hospitalized for COPD (54). So, the results may apply especially to male COPD patients in similar settings, but future research with more women is needed to explore if there are differences between the sexes.

### 4.1 Limitations

The cross-sectional design of this study precludes establishing a causal relationship between variables. Because the sample consisted of individuals diagnosed with COPD and was from a single center, the generalizability of the results is limited. Another limitation is the predominance of male participants, which likely reflects regional epidemiological characteristics of COPD in Türkiye, where hospital admissions and hospitalization rates are higher among men, particularly in the Black Sea region (54). This sex imbalance may limit the generalizability of the findings, particularly regarding psychosocial variables such as depression, anxiety, emotion regulation, and perceived social support. Furthermore, the use of self-report measures for psychosocial variables and quality of life carries a risk of response bias. Although major clinical covariates such as age, sex, comorbidity status, smoking status, and GOLD stage were included in the mediation models, residual confounding cannot be entirely excluded due to the cross-sectional design.

## 5 Conclusion

This study demonstrated that psychosocial processes, along with respiratory and clinical parameters, play a significant role in quality of life in COPD. As disease severity increases, so does the psychological burden associated with depression, anxiety, stress, and difficulty regulating emotions. Our study found that DER mediates the effect of symptom burden on physical quality of life. Furthermore, perceived social support was found to positively affect physical quality of life by reducing depressive symptoms. Moreover, stress levels have been shown to negatively affect psychological quality of life by decreasing perceived social support. The fact that all these psychosocial effects were found to be independent of COPD stage indicates that these factors are not the result of disease progression but relatively independent determinants of quality of life. Therefore, in COPD management, psychosocial mechanisms should be strengthened alongside improvements in respiratory and clinical parameters. Adopting a multidisciplinary approach to COPD can improve patients’ overall quality of life by improving both their physical and psychological well-being. Further elucidating these relationships through intervention-based designs and longitudinal follow-up in future studies will improve overall outcomes and quality of life in Patients with COPD.

## Data Availability

The raw data supporting the conclusions of this article will be made available by the authors, without undue reservation.
